# Retraction Note: Regulatory effects of glycyrrhizae radix extract on DSS-induced ulcerative colitis

**DOI:** 10.1186/s12906-023-04051-3

**Published:** 2023-06-27

**Authors:** Yong-Deok Jeon, Keuk-Soo Bang, Min-Kyoung Shin, Jong-Hyun Lee, Young-Nam Chang, Jong-Sik Jin

**Affiliations:** 1 Department of Oriental Medicine Resources, Chonbuk National University, 79 Gobongro, Iksan, Jeollabuk-do 570-752 Republic of Korea; 2 Department of Pharmacy, College of Pharmacy, Dongduk Woman’s University, 23-1 Wolgok- Dong, SungBuk-Gu, Seoul, Republic of Korea

**Retraction Note: BMC Complement Med Ther 16, 459 (2016)**

10.1186/s12906-016-1390-8

The Editor has retracted this article because of significant concerns regarding Figs. 2 and 4 A presented in this work, which question the integrity of the data. Further investigation found substantial text overlap with two other publications by the same authors [1, 2]. The authors did not provide original data upon the journal’s request. The corresponding author Jong-Sik Jin stated that serious mistakes have been found in data processing and that the study was under investigation by the authors’ institution. The Editor therefore no longer has confidence in the integrity of the data in this article. Yong-Deok Jeon, Keuk Soo Bang and Jong-Sik Jin agree with this retraction but not with the wording of the retraction notice. Min-Kyoung Shin, Jong-Hyun Lee and Young-Nam Chang have not responded to correspondence from the Editor about this retraction.

## References

[1] Kim D-S, Kim S-J, Kim M-C, Jeon Y-D. Jae-young Um, Seung-Heon Hong, The Therapeutic Effect of Chelidonic Acid on Ulcerative Colitis, Biological and Pharmaceutical Bulletin, 2012, Volume 35, Issue 5, Pages 666–671, Released on J-STAGE May 01, 2012, Online ISSN 1347–5215, Print ISSN 0918–6158.10.1248/bpb.35.666. 10.1248/bpb.35.666 22687399

[2] Kim D-S, Ko J-H, Jeon Y-D, Han Y-H, Kim H-J, Poudel A, Jung H-J, Ku S-K, Kim S-J, Park S-H, Park J-H, Choi B-M, Park S-J, Um J-Y. Seung-Heon Hong, “Ixeris dentata NAKAI Reduces Clinical Score and HIF-1 Expression in Experimental Colitis in Mice”, Evidence-Based Complementary and Alternative Medicine, vol. 2013, Article ID 671281, 9 pages, 2013. 10.1155/2013/671281. 10.1155/2013/671281 PMC3782128 24194783

